# Decision Support System Proposal for Medical Evacuations in Military Operations †

**DOI:** 10.3390/s23115144

**Published:** 2023-05-28

**Authors:** Piotr Lubkowski, Jaroslaw Krygier, Tadeusz Sondej, Andrzej P. Dobrowolski, Lukasz Apiecionek, Wojciech Znaniecki, Pawel Oskwarek

**Affiliations:** 1 Faculty of Electronics, Military University of Technology, Gen. Sylwestra Kaliskiego 2, 00-908 Warsaw, Poland; jaroslaw.krygier@wat.edu.pl (J.K.); tadeusz.sondej@wat.edu.pl (T.S.); andrzej.dobrowolski@wat.edu.pl (A.P.D.); 2 Institute of Computer Science, Kazimierz Wielki University, Jana Karola Chodkiewicza 30, 85-064 Bydgoszcz, Poland; lapiecionek@teldat.com.pl; 3 Teldat Sp. z o.o. sp.k, Cicha 19, 85-650 Bydgoszcz, Poland; wznaniecki@teldat.com.pl; 4 Military Institute of Medicine–National Research Institute, Szaserów 128, 04-141 Warsaw, Poland; poskwarek@wim.mil.pl

**Keywords:** enterprise architecture, decision support system, medical evacuation, vital signs measurement, biomedical signals measurement

## Abstract

The area of military operations is a big challenge for medical support. A particularly important factor that allows medical services to react quickly in the case of mass casualties is the ability to rapidly evacuation of wounded soldiers from a battlefield. To meet this requirement, an effective medical evacuation system is essential. The paper presented the architecture of the electronically supported decision support system for medical evacuation during military operations. The system can also be used by other services such as police or fire service. The system meets the requirements for tactical combat casualty care procedures and is composed of following elements: measurement subsystem, data transmission subsystem and analysis and inference subsystem. The system, based on the continuous monitoring of selected soldiers’ vital signs and biomedical signals, automatically proposes a medical segregation of wounded soldiers (medical triage). The information on the triage was visualized using the Headquarters Management System for medical personnel (first responders, medical officers, medical evacuation groups) and for commanders, if required. All elements of the architecture were described in the paper.

## 1. Introduction

Medical evacuation, also abbreviated as MEDEVAC, is a set of activities provided by medical personnel to evacuate wounded soldiers or injured patients from a battlefield or from a scene of accident. The first phase of the evacuation process is devoted to a life-saving medical care of the patients at the place of accident and then to en route care provided by medical personnel in emergency medical service vehicles or aeromedical helicopters. In typical situations, the emergency service personnel reacts after receiving information on an accident coming directly form injured persons or from witnesses of the accident. The decision on medical transport of the patients to medical facilities is taken by the personnel of an ambulance (medical rescuers) after the first aid provided at the place of accident. The situation can be more complicated if we have accidents with many casualties with different levels of severity. Often, because of lack of the resources, the medical personnel must decide which patient must be aided firstly and which can wait. In the medicine, such selection is known as a triage. 

Thus, the first triage can be performed by the medical personnel at the scene of accident to decide about the order of patients who have to be evacuated to the hospital firstly and who can be evacuated subsequently. The triage is, in particular, required on the battlefield where many wounded soldiers can suddenly appear during military action, but also at peacetime during accidents with mass casualties. To effectively support the decision on the triage, and to decrease the time of medical evacuation, medical personnel should be equipped with information on the gravity of wounds and patients conditions even before an arrival to the scene of accident. To do so, an effective decision support system for medical evacuation (DSS-MEDEVAC) is needed. The authors of this paper propose such a system for military use, elements of which might be used by other services such as police, fire service or organized rescue groups. The system was developed from scratch and it is proposed for armed forces supported by command and control information systems. The main goal of the DSS-MEDEVAC system is to monitor the health parameters of the soldiers during military actions, process these parameters and refer the decision on suggested military triage to the military personnel in case of identified life and health risks of the soldiers. Simultaneously, the information on potential medical problems can be passed to the commanders to support their decision during military operations. Based on the information from the DSS-MEDEVAC, the medical personnel that is responsible for tactical evacuation care, is able to react faster on the situation on the battlefield having current information on the suggested triage and the casualties health. This information supports the medical officers in the decision process on medical evacuations. Additionally, the information is passed to the medical evacuation vehicles or helicopters to constantly support the evacuation personnel. 

The DSS-MEDEVAC system is composed of the health monitoring sensors integrated with the soldiers’ uniform and personal equipment, communication module responsible for reliable medical data transmission, decision support module responsible both for processing the data received from the sensors of each soldier and for taking the initial decision on the triage. This information supports the medical officer, who is able to take a final decision on medical evacuation and medical care before and during the evacuation. Additionally, the medical personnel of the evacuation vehicle can change the decision suggested by the system after the first aid at the battlefield or during the en route care. The new data are processed by the DSS-MEDEVAC system and are automatically conveyed to the deployed or fixed medical facilities (decision support center, evacuation points, field hospital). The DSS-MEDEVAC system automatically fills out an electronic TCCC card (tactical combat casualty care card) according to the information provided by its medical sensors. This electronic card continuously provides current patient data trends and can replace the paper TCCC card used at present, which is attached to wounded soldiers for evacuation and can only be updated when possible. Thus, the information on the casualties can be immediately consumed by the personnel of medical facilities to prepare required medical resources. 

This paper focused on the description of the DSS-MEDEVAC system architecture, general explanation of the system components and on the presentation of the first laboratory tests of these components. The DSS-MEDEVAC is a pre-verified system proposed for implementation in regular military units. The system is planned to be applied in Polish Armed Forces after field tests. 

We can distinguish following advantages of our system:

- It supports military medical personnel in automatic information on current health of the soldiers during military operations;
- It allows a quick reaction of medical evacuation groups in case of mass casualties on a battlefield;
- It supports an automatically pre-hospital triage of wounded soldiers;
- It supports updating the electronic tactical combat casualty care cards.

The limitation of the system is that it needs a working communication system to periodically transmit the measurement data from the sensors to the inference and analysis subsystem. Thus, it cannot operate during so-called radio silence.

The paper is organized as follows. The next section describes related work regarding similar health monitoring systems both for regular and dedicated medicine. In Section 3, the proposed DSS-MEDEVAC system architecture is clarified. The elements of the system are described from Section 4, Section 5, Section 6 and Section 7. The last section concludes the paper.

## 2. Related Work

Remote health monitoring is not a new technical problem. Many solutions were proposed for wearable health monitoring sensor systems over the past several decades [1]. Additionally, many ready-for-use products can be found on the market [2,3,4,5]. They are mainly composed of the measurement unit constantly or periodically used by the patients in their home to measure and monitor selected vital signs, data transmission unit (mostly smartphone or wi-fi router) and health monitoring center, allowing visualization of threats in health alarms, measured vital signs and basic biomedical signals. These solutions are gaining importance in the age of so-called telemedicine, where a doctor can quickly react on health problems of his patients. As mentioned in the introduction, a remote health monitoring is leveraged mostly by diagnosed patients. The solutions are also equipped with the automatic call to the emergency service in case of identified critical alarms. Unfortunately, regular health monitoring systems cannot be directly used by soldiers during military operations to constantly monitor their critical vital signs. 

Remote health monitoring solutions currently available on the market are based on the so-called wearable devices and have many limitations important from a military viewpoint, but importantly, they are proprietary solutions and, thus, cannot be easily integrated with military headquarters management systems. One of the interesting systems is Zephyr BioHarness [6], which is also proposed by the manufacturer for limited military use. Unfortunately, it does not allow measuring all the vital signs we assumed to be used in our system (i.e., blood pressure, oxygen saturation), but also its communication range and technology is not acceptable for tactical operations (up to 274 m with Zephyr ECHO Gateway communication module).

An Interesting approach to remote monitoring of footballers’ health is presented in [7], where movement responses were registered by GPS (global positioning system) units and conveyed to the analysis in order to optimize sportspersons’ performance depending on the speed intensity. To the best of our knowledge, there are no studies that used similar health monitoring methods to assess combat effectiveness or the ability to avoid injury by soldiers during military actions. We also do not assume that our system should support such a capability. Avoiding injury by the soldiers is very important, but it is not the main priority, at the theater of military operations. Therefore, activity of soldiers should mainly be optimized to reach military tasks.

The CRI (compensatory reserve index) proposed in [8] is a novel vital sign parameter that uses features derived from machine learning of the arterial pulse wave or photoplethysmography (PPG). In our system, we also measure the PPG signal and we have the ability to remotely send it to the analysis and inference subsystem. As indicated by the authors of [8], the CRI parameter can be useful to identify the bleeding trauma soldiers which we will try to consider in the next version of our system.

In turn, in the article [9], the authors explored three parameters (SI—shock index, PP—pulse pressure and ROX index) in terms of their usefulness in pathophysiologic categorization of patients in a hospital. However, these are hospital conditions, and the results of the work refer to mortality. The main role of our system is to support a decision on the soldiers’ evacuation from the battlefield. Nevertheless, the above parameters were calculated on the basis of the main vital signs, i.e., heart rate, blood pressure, oxygen saturation and respiratory rate, which are also measured in our system.

A number of solutions are also dedicated specifically to support military medical care. One such solution is the NATO First Responder (NFR) application developed by the U.S. Defense Health Agency for documenting injuries and care for casualties while they are being resupplied on the battlefield [10]. The application enables the preparation of reports to be forwarded to medical services before a wounded soldier is evacuated to fixed medical facilities. Communication in this case is based on the mobile networks. In the absence of access to cellular network resources, information about patients can be shared using near field communication (NFC) tags. The aforementioned solution complies with NATO standards for the evacuation and medical protection of soldiers on the battlefield. However, it is worth noting that the NFR does not support remote monitoring of wounded soldiers.

The OpenAhlta is an open source version of the U.S. Department of Defense battlefield electronic medical record system (AHLTA-Theater) and operates using NFR application [11]. It uses Health Level Seven (HL7) standards for transferring clinical and administrative data. Unfortunately, it cannot be a base system for remote health monitoring of the soldiers on the battlefield.

The BATDOK (battlefield-assisted trauma distributed observation kit) created and owned by the U.S. Air Force Research Laboratory’s 711th Human Performance Wing is a software that can be run on a smartphone or other mobile devices to enable medics to wirelessly monitor multiple patients’ vitals at the point-of-injury [12]. The vital signs values can be delivered to the application by all available methods, starting form a measurement using accessible specialized medical devices and finishing on an observation of wounded soldiers by first responders. This solution is conceptually very similar to our system, but it is not integrated with sensors to constantly monitor health of soldiers even before injury. It is also not integrated with a battlefield management system to constantly support medical services on different levels of command.

In addition to remote health monitoring systems, some triage supporting algorithms were elaborated. Most of them are just procedures that the rescuers must apply to effectively provide a medical assistance at a scene of an incident. An example of such an algorithm is START (simple triage and rapid treatment) [13] or SALT (sort, assess, life-saving interventions, treatment and/or transport) [14], The START algorithm supports first responders, allowing them to triage multiple victims in a very short time, based on three observations, i.e., respiration, perfusion and mental status. Responders assign patients to four categories: deceased, immediate, delayed and minor. Triage tags are used (mostly physical marking) to distinguish patients sorted to separate categories. Similarly to the START, the SALT is a four-step process helping first responders to manage mass casualty incidents. It also proposes the tags to segregate the patients (dead, immediate, expectant, delayed and minimal). Both START and SALT are relatively simple triage algorithm based on vary basic observations; thus, they often are used at the first stage of segregation process. More advanced systems such as TEWS (triage early warning system) [15] or NEWS2 (national early warning score 2) [16] require more measurements (i.e., blood pressure, heart rate, oxygen saturation and others) to triage casualties. 

The authors of [17], in their paper, discussed one of the triage methods that can be successfully applied in regular emergency services. This method refers to the triage of the patients who subsequently required hospital admission and who were likely to die within 24 h. Since available criteria pointed out in the cited paper can be used for selecting soldiers who can quicker be recovered and returned to military actions, we propose nearly the same approach in our system for so-called reverse triage, but we should remember that tactical operations often preclude rapid medical assistance and evacuation, where rescuers often cannot immediately reach the point of injury and evacuation process is delayed (the military priorities are more important than medical evacuation of wounded soldiers during military operations). Having the constant monitoring system that is based not only on the measurement of respiratory rate or oxygen saturation, we can offer the military medical personnel means for fast reaction even before arriving to the point of injury or even during lack of medical assistance. The medical evacuation personnel can remotely instruct (using military radios) first responders (neighboring soldiers which can help wounded soldiers) based on the information coming from our system. Additionally, the basic vital signs that we monitor only suggest the initial triage, which must be confirmed by first responders (commander or the soldiers which are able to prepare and send requests for medical evacuation), where easily available criteria pointed out in the cited paper are applied. In addition to the information on the initial triage, our system allows sending certain biomedical signals that are generated at the request of medical personnel alerted by initial triage. All the above actions can be initiated automatically owing to constant monitoring of selected vital signs. However, it should be noted that there are also some limitations of our system. This situation will take place during radio silence (specific military situations where radios cannot transmit data for security reasons), but this limitation is valid for all remote monitoring systems.

Taking into account the mentioned triage support systems, the authors prepared a set of capabilities which the DSS-MEDEVAC system have to achieve. The required capabilities of the DSS-MEDEVAC are described in Section 3.

## 3. Proposed Architecture and Requirements of the Decision Support System for Medical Evacuation (DSS-MEDEVAC)

A system that should support medical personnel during military operations will differ from typical healthcare telemonitoring systems, although the goal of such systems is similar in both cases; they must measure the relevant vital signs of the patients/soldiers/firefighters, and transfer the data to the health monitoring center in order to react faster on critical health events. However, in a military, tactical environment (on the battlefield), the main priority is to achieve the military goal. Therefore, the rapid recovery of the soldiers to use them again in combat is a crucial task for medical personnel. To cope with this task, a reverse triage is often used during pre-hospital medical care. 

To help military medical personnel to make decisions on fast evacuation of the wounded soldiers from a battlefield, constant health monitoring is required, not focusing on specific illness of a soldier (but taking into account personal health parameters). Such constant monitoring requires not only a reliable measurement of human vital signs, but also should take into account the specific battlefield conditions (the soldiers can run, drop, can be overstressed, and eventually can be slightly or severely wounded, or can suffer death on a battlefield). Taking such conditions into account, the soldiers’ health monitoring system will differ from the health monitoring solutions available on the civilian market. The authors prepared the architecture of such system, focusing on supporting medical personnel on a battlefield, especially in terms of faster triage before and during medical evacuation process. The abbreviations used during system description are collected in Table 1.

### 3.1. System Requirements

The architecture of DSS-MEDEVAC system dedicated for military operations was prepared based on the NATO Architecture Framework version 4 (NAFv4) [18], which is a standard for developing and describing architectures for both military and business use. The NAFv4 supports preparation of different viewpoints of the developed system, including concept of the system (C group of viewpoints), service specifications (S group of viewpoints), logical specifications (L group of viewpoints), physical resource specifications (P group of viewpoints) and architecture foundation (A group of viewpoints). 

In this section, the main viewpoints of the DSS-MEDEVAC system are described, i.e., Capability Taxonomy (C1), Enterprise Vision (C2), Capability Dependencies (C3), Service Taxonomy (S1), Node Types (L1) and Logical Scenario (L2).

**Figure 1:**
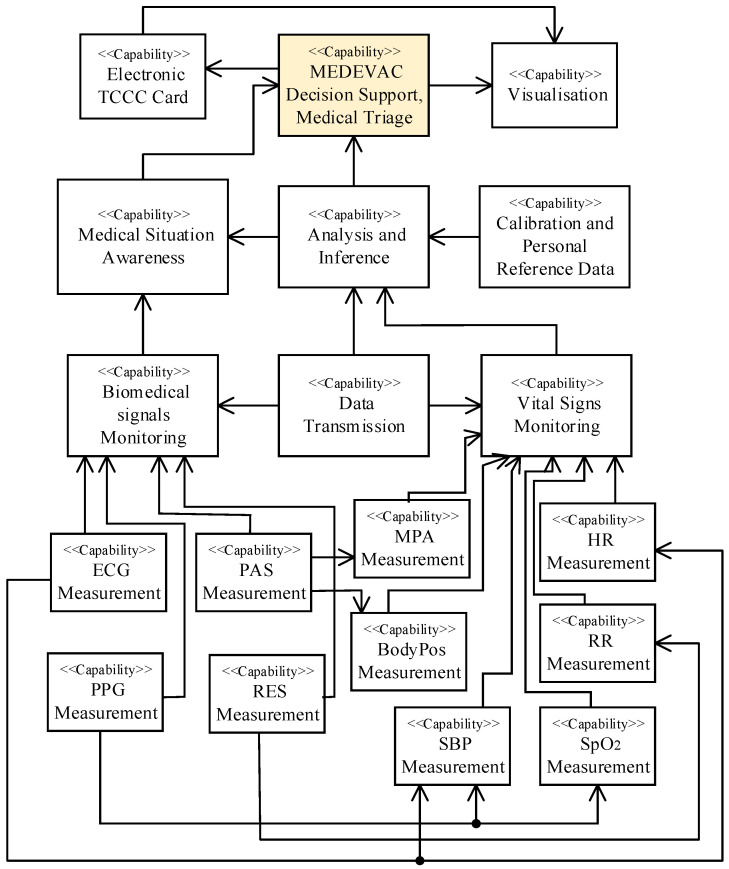
DSS-MEDEVAC capability taxonomy and capability dependencies.

To reflect the goal of the DSS-MEDEVAC system, the main system capabilities are defined. Figure 1 shows proposed the simplified system Capability Taxonomy (C1) and Capability Dependencies (C3) viewpoints.

It was assumed that the system must present to the medical personnel and selected commanders the current information on the soldier’s vital signs and initial decision on the triage, in the case of identified (detected) life or health threats (medical situation awareness capability—MSAC). Additionally, the system must present current location of each soldier to help organize the medical evacuation. The system must be capable of measuring, registering and processing the required human vital signs (vital signs monitoring capability) and reliably send the results of the measures (data transmission capability—DTC) to the analysis and inference module. Then, the system must effectively process the measured data to propose initial decision of the triage (analysis and inference capability—AIC). 

After detailed analysis, it was decided that the main goal of the system can be achieved by supplying the AIC by following human vital signs: heart rate (HR), respiratory rate (RR), oxygen saturation (SpO_2_), mean physical activity (MPA), body position (BodyPos) and systolic blood pressure (SBP). 

The set of vital signs was selected on the basis of four factors, i.e.:

- Related work analysis on proposed solutions supporting the triage during mass casualty incidents (for example: [19,20]);
- Related work analysis on supporting remote health monitoring during disaster-induced mass casualty incidents by Internet of Thigs (IoT) solutions (for example: [21]);
- Analysis of similar solutions dedicated for the military (for example: [10,11,12]);
- Personal experience of first responders and pre-hospital medical personnel on medical evacuation during military operations.

The authors of [21] proposed a real-time monitoring electronic triage tag system for improving survival rate in disaster-induced mass casualty incidents—the goals of this system were, therefore, similar to the goals of our system. They also proposed vital signs that can be simply measurable during pre-hospital triage, i.e., body temperature, heart rate, blood pressure, respiratory rate, oxygen saturation, capillary refill, consciousness and electrocardiogram. The authors of [21] also argued that vital signs such as heart rate, blood pressure, respiratory rate and electrocardiogram are considered to be the most important factors by field rescuers. Since the triage in our system is based on the simple triage and rapid treatment (START) procedures, we also proposed a set of vital signs that can be constantly, autonomously, non-invasively and, importantly, simply measurable during military operations (for example, SpO_2_ and HR), even without a specialized medical equipment (for example, RR). 

The selection of the vital signs for our system also resulted from the analysis of other triage systems (procedures). Most triage systems are based on a simple test of consciousness using ACVPU (Alert, Confusion, Voice, Pain, Unresponsive) scale, presence of a pulse on the radial artery and the number of respirations, but in more advanced triage systems, SpO_2_, SBP, HR are also measured. In [19], an extensive analysis of the currently functioning triage systems is shown. The authors of [20] also performed an analysis on the triage systems and useful vital signs relating to the mass-casualty bioterrorism. The vital signs we selected for our system are often the main variables taken into account in analyzed triage systems. The analysis led us to the conclusion that we need a minimal set of vital signs that is able to support rapid remote inference about a soldier’s health status, taking into account strong military restrictions and procedures.

The AIC should also be supplied by the calibration and reference data, specific for each soldier (calibration and personal reference data-loading capability). Additionally, the system must enable the medical officers or rescuers to remotely observe required biomedical signals of a selected soldier what can help them to prepare in advance required medical equipment and medications, and to update the decision on the triage, if required (biomedical signals monitoring capability). Following biomedical signals of the soldier being assumed to be monitored remotely on demand: electrocardiogram (ECG), photoplethysmogram (PPG), physical activity signal (PAS) and respiratory rate signal (RES). The registered vital signs of the wounded soldiers must be automatically passed to the electronic tactical combat casualty care card, which must be immediately available in the system for the medical personnel involved in the soldiers’ treatment (Electronic TCCC Card Filling Capability). Both the TCCC card and the initial decision on the triage must be immediately available for viewing in ‘real time’ in the headquarters management system (HMS) used by the Armed Forces during military operations (visualization capability).

### 3.2. Realization of System Requirements

**Figure 2:**
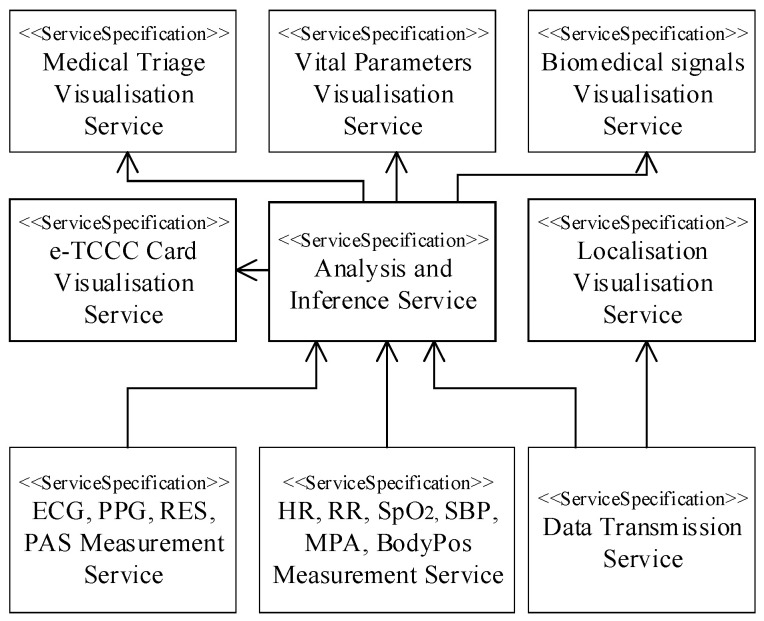
DSS-MEDEVAC service taxonomy viewpoint.

A service taxonomy viewpoint (S1) is shown in Figure 2. The services offered by the DSS-MEDEVAC system reflects the capabilities that are required (shown in Figure 1). The main service group is responsible for visualization of the information that is concerned with the triage decision, soldier’s localization, vital signs, biomedical signals and electronic TCCC card. The important service required by the system is also the data transmission service, which ensures reliable data distribution over all the players in the system.

**Figure 3:**
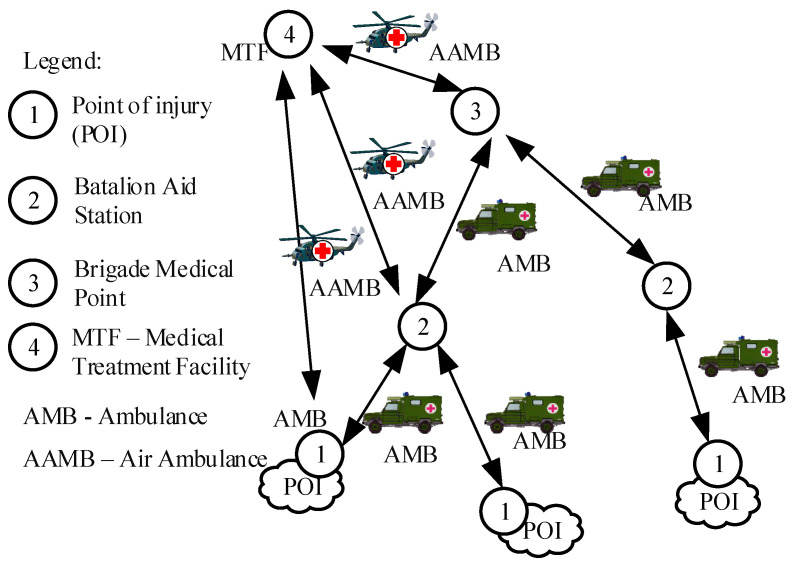
DSS-MEDEVAC operational (enterprise) viewpoint.

The operational viewpoint of the medical evacuation actions where the DSS-MEDEVAC system is planned to be employed is shown in Figure 3. Wounded soldiers are firstly evacuated to a secure area at the battlefield (if it is possible) to give them the first aid. Let us name this area the point of injury, where the first professional medical assistance can be delivered by the medical personnel (military ambulance—AMB or air ambulance—AAMB) after reaching this area. The sensors of DSS-MEDEVAC system should firstly react on the life problems of the casualties; thus, medical personnel can be supported by important life parameters at the early stage of the evacuation process. The initial triage is also suggested immediately; thus, the required medical resources can be involved in the medical evacuation process. Additionally, current biomedical signal of the wounded soldiers can be observed before the evacuation and during the en route care (supporting the professional medical instruments). The upper levels of the military evacuation pointed out in Figure 3 can exploit both the electronic TCCC card and the information on the initial and updated triage and the current conditions of wounded soldiers. The operational viewpoint shows the main players involved in the DSS-MEDEVAC systems.

**Figure 4:**
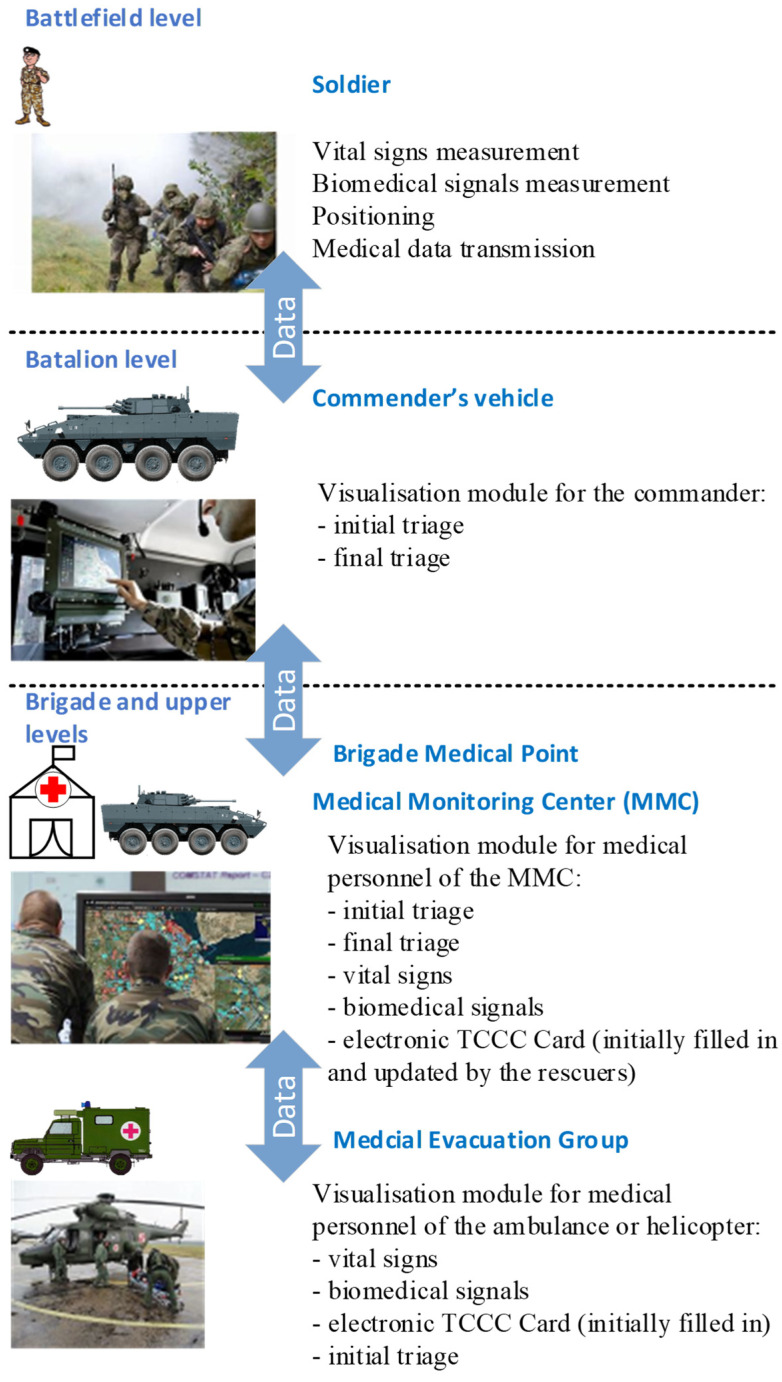
DSS-MEDEVAC system general viewpoint.

Figure 4 clarifies the DSS-MEDEVAC system general viewpoint. At the single soldier level, a set of bio-sensors have to be employed to measure both the vital signs and biomedical signals, which are important for the initial triage preparation and from all medical evacuation process point of view (indicated and explained in the capability viewpoint).

The general information on the soldier’s life-condition (initial and final triage) is visualized by the battlefield management system (BMS) to the direct commander, supporting the current situation awareness. The set of vital signs are constantly sent using the prepared communication protocol via the communication network to the medical monitoring center (MMC), where the data are analyzed to propose the initial and final decision on the triage. This information is then passed to the visualization module available to the medical personnel of the MCC, which can take a final decision on medical evacuation. The medical personnel can also observe the required biomedical signals of the wounded soldiers to additionally support the decision on evacuation or to advice remotely on the first aid. The same information appears in the visualization module of the medical evacuation groups (MEG)—ambulances. The medical personnel of the MEG can change the decision on the triage after direct medical actions at the point of injury. Initially filled in the electronic TCCC card, associated with each wounded soldier, is also updated by the rescuers and is immediately available for medical personnel at each level of the evacuation facilities.

**Figure 5:**
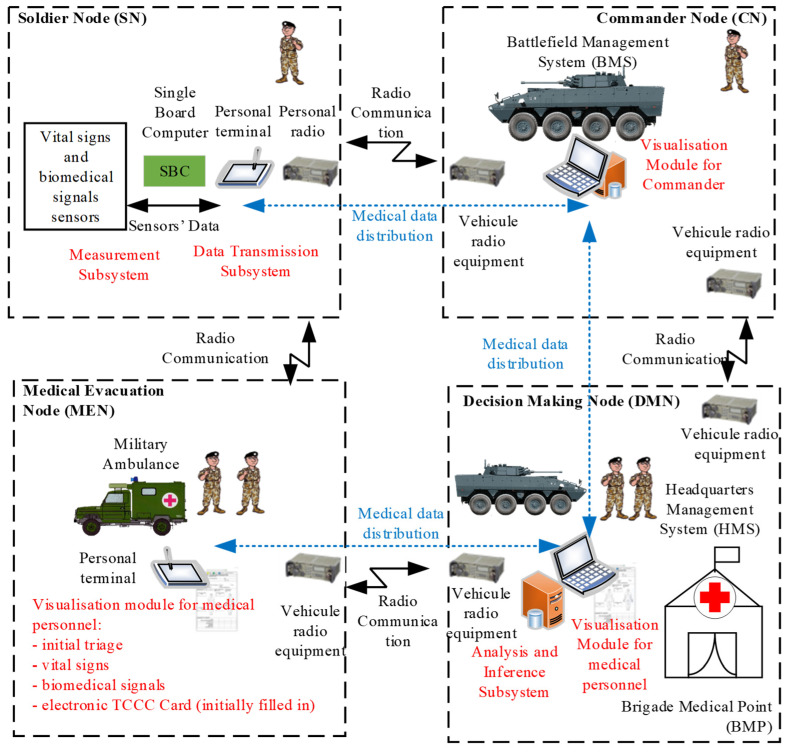
DSS-MEDEVAC logical viewpoint—nodes types viewpoint.

The logical viewpoint of the DSS-MEDEVAC system is presented in Figure 5. Three main nodes are distinguished in the system: Soldier Node (SN), Commander Node (CN), Decision Making Node (DMN) and Medical Evacuation Node (MEN).

The SN is composed of a body area bio-sensors’ network (BAN), a single board computer (SBC), a personal terminal and a personal radio. The BAN is a network integrated with personal equipment of the soldier (including military uniform, helmet and underwear). The SBC is designed to perform initial processing of biomedical signals and real-time computing of vital signs and to refer the processed data to the personal terminal via a developed communication protocol. The personal terminal is a tablet-based military computer, which constitute the gateway to the tactical data transmission system. It is also assumed that the BAN and, particularly, the SBC can monitor and store the history of the vital signs of the soldier even if the other equipment is inaccessible. These values can be accessible later on if required. The tactical data transmission system is a part of the BMS and HMS systems. Its main role is to reliably distribute the medical data over all elements of the DSS-MEDEVAC system using tactical radio networks (personal radios, vehicular radio equipment, deployed and fixed communication infrastructure).

The CN role is to collect the medical data transmitted by subordinates and to send them to the DMN. Based on the data received from the CNs, the DMN node performs the advanced analysis and inference and suggests the decision on the triage. The decision is passed to the visualization modules located in commander nodes, medical evacuation nodes and other medical facilities involved in the medical evacuation actions. The medical data are distributed between the nodes of the DSS-MEDEVAC using the tactical data transmission system. The information on the triage, vital signs and biomedical signals is visualized both to the medical personnel and the commanders with the use of BMS and HMS systems. The medical evacuation node (ambulance, aeromedical helicopter), receives the current decision on the triage and initially filled in the electronic TCCC card to support the rescuers. The MEN is also the source of the information on the final decisions which are passed to the DMN and distributed over the system.

The main subsystems of the DSS-MEDEVAC system, which reflect the components of the logical viewpoint presented in Figure 5, are described in the next sections.

## 4. Measurement Subsystem

**Figure 6:**
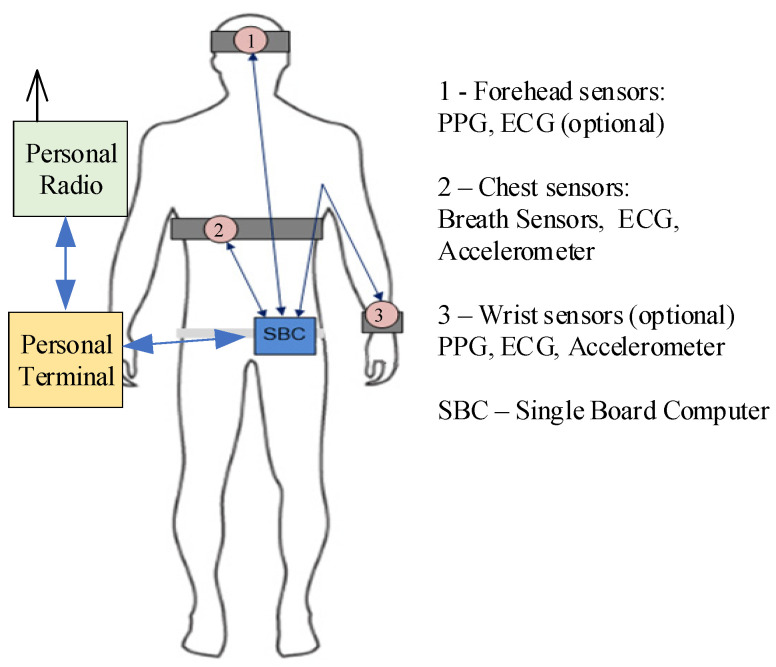
General architecture of the measurement subsystem of the DSS-MEDEVAC.

Using different kind of sensors for measurement of the biomedical signals and vital signs in a relatively static body position is well described in the literature. In the DSS-MEDEVAC system, we assume difficult measurement conditions, where some sensors can be destroyed or disconnected and the analysis of the received data should predict such situations. Moreover, we should assume that commonly known wireless transmission techniques dedicated for sensor networks (i.e., Bluetooth, ZigBee, ANT or Near Field Communications) cannot be applied during military operations, especially because of intentional jamming of radio transmissions. All these considerations lead to the decision on a set of health parameters that should be monitored, initially processed and sent to the analysis and inference subsystem, as well as to the decision on the communication technique that prevents data transmission from jamming. Additionally, wearable sensors should be integrated with a personal soldiers’ equipment (uniform, underwear, communication equipment). A simplified architecture of the measurement subsystem is shown in Figure 6. 

Wearable sensors, constructed by the authors of this paper, are located on the forehead, chest and wrist (optional sensor) of a soldier. They are integrated with a helmet and with an uniform. A single board computer (SBC) based on high-performance arm cortex-M7 microcontroller with low power consumption is responsible for data acquisition from sensors, initial processing of the measured signals, real-time computing of vital signs and communication with personal terminal. To ensure jamming protection, data are transferred to the SBC using wired communication, where wires are to be integrated with an uniform. Both the SBC and sensors are powered by a personal terminal (as a main energy source) or by an internal SBC battery (in a stand-by or emergency mode). It is assumed that the personal terminal is able to deliver required energy to the measurement subsystem during the military mission. The data are sent to other parts of the system via a personal radio which ensures secure and robust tactical communication (even under hostile jamming).

**Figure 7:**
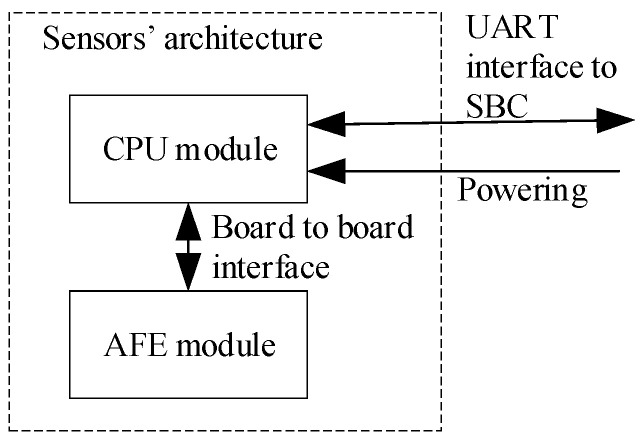
DSS-MEDEVAC sensors’ architecture.

In general, a large set of biomedical signals and vital signs can be measured to support remote health monitoring [2,22]. Nevertheless, we do not have to use all of them to support a decision on the triage in military operations (or in similar actions). Taking into account the required capabilities of the DSS-MEDEVAC system, specific military conditions, and after deep analysis of the military health care and evacuation procedures [23] during military actions, we decided to measure biomedical signals and vital signs pointed out in Figure 1. These signs are supported by information on soldier’s motion and body position (by using of an accelerometer) as shown in Figure 6. On the current stage of the system development, authors prepared a testbed with all required sensors, where each sensor is composed of two main components: CPU (central processing unit) and AFE (analog front end) modules, shown in Figure 7. A sensor is able to communicate with the SBC via two-wires UART (universal asynchronous receiver-transmitter) interface. Such a modular architecture of the sensors allows using dedicated AFE for each sensor with the same CPU module.

**Figure 8:**
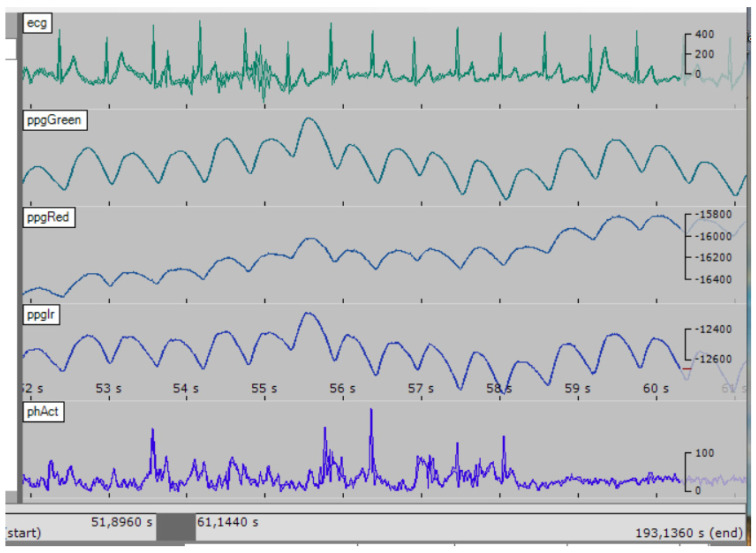
Example signals plot measured by the AFE modules of the sensors (“,” means decimal separator).

As shown in Figure 6, measurement subsystem of the DSS-MEDEVAC contains three sensors measuring biomedical signals. These signals are integrated in the SBC computer; therefore, it is important to ensure synchronization of the measurement of these signals. This is especially important when calculating the SBP parameter. The SBP will be measured continuously, cuffless and non-invasive, based on the propagation time (PAT—Pulse Arrival Time or PTT—Pulse Transit Time) of the pulse wave calculated from the ECG/PPG signals [24,25,26,27]. In our measurement subsystem, shown in Figure 6, we proposed a distributed, wired system of sensors synchronized by the SBC. To control the sensors, the SBC periodically sends requests to each sensor. Each of the sensors must respond to the request (i.e., it sends signals samples) in a fixed time, less than the highest sampling period of the requested signal (e.g., for ECG the response time must be less than 4 ms). In our sensors, we used STM32L5 series of the microcontrollers that have nested vectored interrupt controller (NVIC), allowing preparing up to eight interrupt priorities. The requests received by the sensors from the SBC (i.e., interrupt of receiving a byte from the UART interface) have an appropriate high priority. Thanks to this, the sensor’s response (i.e., starts data transmission via UART) time is below 100 µs. Figure 8 shows that developed AFE modules are able to measure and send the ECG and PPG signals, which are then sent to the SBC.

The SignalPlant [28] software was used to display the data in Figure 8. It facilitates viewing multiple signals with the same sampling rate and has many useful functions, including data processing.

## 5. Data Transmission Subsystem

**Figure 9:**
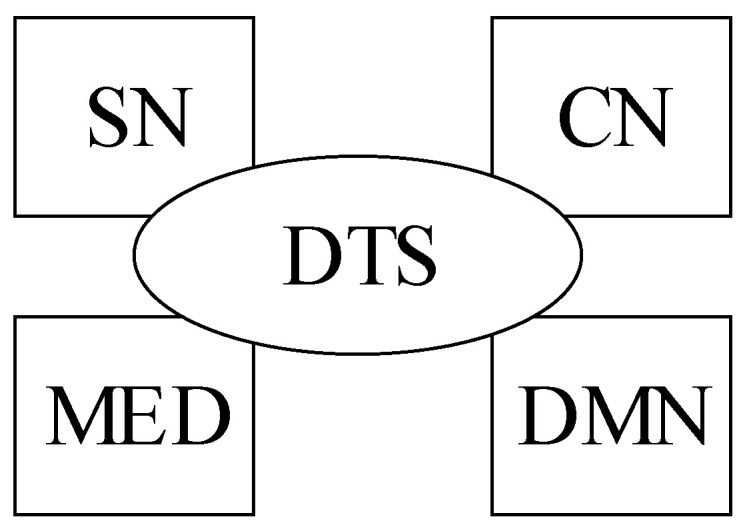
Data Transmission Subsystem as an integrator of the DSS-MEDEVAC nodes.

A role of the data transmission subsystem (DTS) is to provide the data transmission service (pointed out in Figure 2) to the DSS-MEDEVAC system. The DTS integrates SN, CN, DMN and MED nodes, as shown in Figure 9.

Reliable medical data distribution between the nodes are ensured by the headquarter management system (HMS), initially developed to support command, control, communication, intelligence and surveillance (C3IS) capability during military operations [29]. The HMS C3IS system in its basic form enables commanders to make decisions in a timely manner, by acquiring information reported, creating a common picture of the operational situation and exchanging operational information. Thus, the HMS can also be powered by the information form DSS-MEDEVAC to support medical personnel at each level of the military medical care, including military medical evacuation activities (as shown in Figure 4). A core of the DTS is a tactical communication network based on the Internet protocol (IP). The HMS working on the top of the IP-based communication network ensures reliable database updating and replication. In this section, we focused on one part of the DTS, i.e., on the method of data delivery to the HMS.

**Figure 10:**
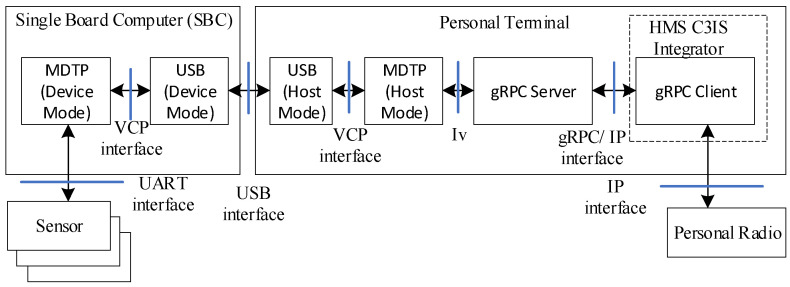
Communication component of the soldier node.

Figure 10 shows a communication component of the soldier node.

As was already mentioned, the sensors are controlled by the SBC via the UART interface. Next, the data are transferred to the personal terminal using the universal serial bus (USB) interface, where the SBC runs the USB driver with a device mode, while the personal terminal uses the USB driver with a host mode. To coordinate the data transfer via the USB interface, a MDTP protocol (DSS-MEDEVAC data transmission protocol) was elaborated and implemented, both in the SBC (MDTP device mode) and in the terminal (MDTP host mode). Virtual COM Ports (VCP) are used by the MDTP drivers to communicate with the USB drivers. The data are sent to/from the HMS C3IS system using the Google Remote Procedure Calls (gRPC) [30], which are transferred by IP packets between the gRPC client and server applications. The gRPC client is a part of the integrator which distributes the data over the DSS-MEDEVAC nodes. The Iv interface is responsible for inter-process communication.

## 6. Analysis and Inference Subsystem

In this section, just general information on the triage algorithm is provided, which can clarify the role of the analysis and inference subsystem (AIS) in the DSS-MEDEVAC system. A heart of the AIS is a decision algorithm segregating wounded soldiers by assigning them following colors: green, yellow, red and black. The first three colors reflect the requirements on the triage defined in the START (simple triage and rapid treatment) procedures. The last one was added to indicate communication problems.

We also proposed the appropriate values of the chance of soldiers’ survival metric based on the assessment of practical cases resulting from experiences of the medical evacuation personnel. These values will be optimized after the implementation of the system in the armed forces based on the lessons learned. At the moment, we verified our assumptions in the initial field tests, which confirmed that all elements of the system can be integrated with the battlefield management system, but for obvious reasons, we were unable to verify it on a real battlefield with a large number of wounded soldiers. Thus, we assumed that system is able to learn and modify its initial attributes.

The green color means that chance of survival of a soldier is equal to 100%. It means that a soldier can indeed be slightly wounded, but no medical assistance is needed. The yellow color is assigned to wounded soldiers who should be evacuated but with the second priority. The red color is reserved for soldiers requiring immediate evacuation (with the first priority). If a heart rate signal (HR) is not received from a soldier’s measurement devices, the black color is assigned. It can mean that communication problem accrued or a soldier is dead (this color requires additional verification by medical personnel or other soldiers). In addition to the colors defined above, the blue color can also be assigned to the seriously wounded soldiers with no (or minimal) chance of survival. This color can only be assigned manually by medical personnel or rescuers from the medical evacuation group, after verification of the soldiers’ health at the point of injury (typically on the battlefield). According to miliary procedures, such soldiers will be evacuated with the last priority (known as revers triage).

The system requirements described in Section 3.1 mandate that AIS should be supplied by four main vital signs to make a decision on the triage, i.e., heart rate (HR), respiratory rate (RR), systolic blood pressure (SBP) and peripheral oxygen saturation (SpO_2_). The algorithm can also be supported by information on the physical activity and body position (estimated by accelerometers). The highest priority in soldiers health assessment, in the context of medical segregation, has the HR. The RR is a parameter that rapidly reacts on the changes in human health condition. The SBP is a vital sign resulting from both HR and RR. The value of this parameter reacts slower on deteriorating patient condition. The SpO_2_ is less credible than previous parameters, since it depends on many factors including those not directly connected with threat to life and health; thus, it should be taken into account in last order to prepare a decision on the triage.

The AIS does not require the biomedical signals which are measured by the measurement subsystem. They are sent on demand directly to the medical personnel located in the medical monitoring center or to the rescuers approaching to the scene of accident or battlefield. Thus, the biomedical signals support the first aid and the final decision on the triage. 

The main triage algorithm was clarified in a pseudocode shown in Algorithm 1. According to it, the (green) color is assigned to the soldiers if all measured parameters are in the ranges of the personalized reference values. The (yellow) color is assigned if a value of at least one parameter is outside the reference range but it does not exceed the critical value (which indicates threat to health). One of the rules of the algorithm is that if value of more important parameter enforced the yellow or red color, the triage color cannot be changed down (i.e., to green or yellow color, respectively). After the analysis, the most important vital sign is RR, then HR, SBP and, finally, SpO_2_. Thus, in such an order, the signs were analyzed in Algorithm 1 (RR from line 1, HR from line 8, SBP from line 23 and SpO_2_ from line 38).

The main algorithm is additionally supplemented by a set of algorithms prepared to assess a chance of survival of wounded soldiers and reacting on lack of some parameters from the sensors (what can be often observed during military actions).

On the basis of the main algorithm, an auxiliary algorithm was defined that determines the function of survival chances. The need to define such a function results from the expectations related to the introduction of software supporting triage. The order of evacuation results from the assigned status, marked with the appropriate color, but within the same color, the value of the function of survival chances may be decisive.

In the case of the so-called tactical reverse [31]) triage—as part of TCCC (tactical combat casualty care)—depending on the tactical situation, it is used when the priority is to regain the ability to perform tactical operations as soon as possible, so the lightest wounded will be rescued first; thus, they will be able to return to a tactical action as soon as possible, to recreate combat capability. This is a specific situation in which the least injured are saved in order to be able to use them again in combat. In this situation, the value of the function of survival chances will also be very helpful. In general, for the rescuer, the more information on the input, the better, and the function of survival chances—colloquially speaking—allows you to assess which of the red ones is potentially “more” red than the rest, which can improve the actions of medics in terms of prioritizing evacuation.

In order to define the chance of survival function, 7200 cases were generated that covered the entire four-dimensional parameter space quite well. These cases were used to train two non-linear SVM (support vector machine) networks [32,33,34] and the final result was a function that assigns the red class a range of 1–50%, the yellow class 51–99% and the green class 100%.
**Algorithm 1** The main triage algorithm implemented in the Analysis and Inference Subsystem.**Input:**  Measured values of RR, HR, SBP and SpO2.  Personalized reference and critical values of the vital signs for each soldier.  Typical reference values:    RRref: 9–20/min    HRref: 50–110/min    SBPref: 100–180 mmHg    SpO2ref: >= 94%  RRctitical, HRcritical, SBPcritical, SpO2Critical **Output:**  Triage **Steps:** 1. Read RR 2. if RR == RRref then 3. | triage <- (Green) 4. else if RR != RRctitical 5. | triage <- (Yellow) 6. else 7.  triage <- (Red) 8. Read HR 9. if triage == (Green) 10. | if HR == HRref then 11. | |  triage <- (Green) 12. | else if HR != HRcritical 13. | |  triage <- (Yellow) 14. | else 15. |  triage <- (Red) 16. else if triage == (Yellow) then 17. | if HR == HRref or HR != HRcritical then 18. | |  triage <- (Yellow) 19. | else 20. |  triage <- (Red) 21. else 22.  triage <- (Red) 23. Read SBP 24. if triage == (Green) 25. | if SBP == SBPref then 26. | |  triage <- (Green) 27. | else if SBP != SBPcritical 28. | |  triage <- (Yellow) 29. | else 30. |  triage <- (Red) 31. else if triage == (Yellow) then 32. | if SBP == SBPref or SBP != SBPcritical then 33. | |  triage <- (Yellow) 34. | else 35. |  triage <- (Red) 36. else 37.  triage <- (Red) 38. Read SpO2 39. if triage == (Green) 40. | if SpO2 == SpO2ref then 41. | |  triage <- (Green) 42. | else if SpO2 != SpO2critical 43. | |  triage <- (Yellow) 44. | else 45. |  triage <- (Red) 46. else if triage == (Yellow) then 47. | if SpO2 == SpO2ref or SpO2 != SpO2critical then 48. | |  triage <- (Yellow) 49. | else 50. |  triage <- (Red) 51. else 52.  triage <- (Red) 53. End of the algorithm

To train the SVM network, we used the data generated by a high-fidelity simulator of vital signs of injured patients. The simulator was validated by health parameters measured in trauma patients. This decision resulted from the lack of a sufficient number of measurements carried out on real patients and were only used to verify our algorithms. However, it should be emphasized that SVM network will be trained during the final operation of the system to optimize its behavior. Red, yellow and green classes ranges should be considered as initial values which are planned to be optimized based on the military lessons learned. 

**Figure 11:**
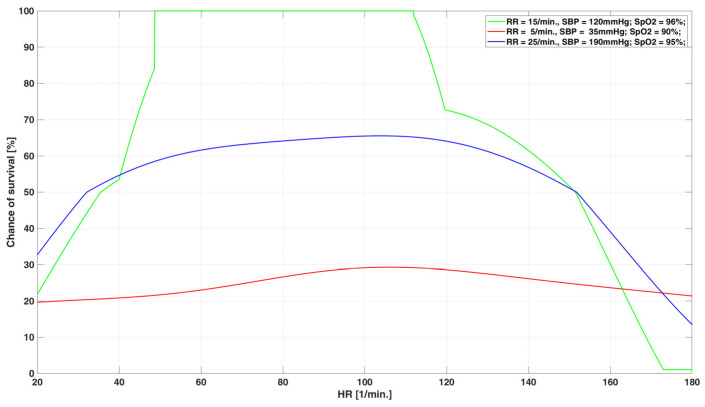
Graph of the chance of survival function for selected values of vital signs.

The graph of chance of survival function for selected parameters is shown in Figure 11. It should be noted that the presented function will also be optimized based on the military lessons learned. We can expect that, in the initial stage of our system’s operation, there may be false alerts, but using learning capability, it should adapt its performance.

It is not uncommon for one or more sensors to be cut off on the battlefield. This may be due to signal cut-off or strong interference preventing the correct determination of the parameter. As a consequence, the following variants of the system operation were used:

1. All four parameters are available. The full algorithm is used and—only in this case—the value of the chance of survival function is calculated. The reliability is 100%.
2. If the SpO_2_ sensor is disconnected, an algorithm using only HR, RR and SBP is applied. The reliability is 90%.
3. If the SBP sensor is disconnected, an algorithm using only HR, RR and SpO_2_ is applied. The reliability is 80%.
4. If the SpO_2_ and SBP sensors are disconnected, an algorithm using only HR and RR is applied. The reliability is 70%.
5. If the RR sensor is disconnected, an algorithm using only HR, SBP and SpO_2_ is applied. The reliability is 80%.
6. If the RR and SpO_2_ sensors are disconnected, an algorithm that uses only HR and SBP is used. The re-liability is 70%.
7. If the RR and SBP sensors are disconnected, an algorithm that uses only HR and SpO_2_ is used. The reliability is 60%.
8. If the RR, SpO_2_, and SBP sensors are disconnected, the HR-only algorithm is used. The reliability is 50%.
9. In the absence of a signal from the HR sensor, the system does not work (regardless of the others). The soldier is marked as “Black”, but it is necessary to confirm it by the medic at the scene.

**Figure 12:**
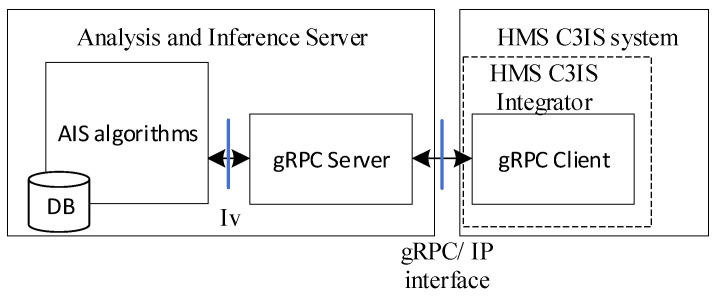
Analysis and Inference Subsystem architecture.

A general architecture of the AIS is shown in Figure 12.

The AIS algorithms are implemented in the Analysis and Inference Server which communicates with the HMS C3IS system via the IP interface using the Google Remote Procedure Calls. The AIS algorithms are supplied by vital signs of each soldier. To coordinate current calculations with historical data, both values of the parameters and the results are stored in a data base (DB). The information on the triage is sent to the HMS C3IS system each time the triage color is changed. The HMS C3IS system is responsible for visualization of the triage to the commander (in limited form, required for command and control), medical personnel or rescuers. 

**Figure 13:**
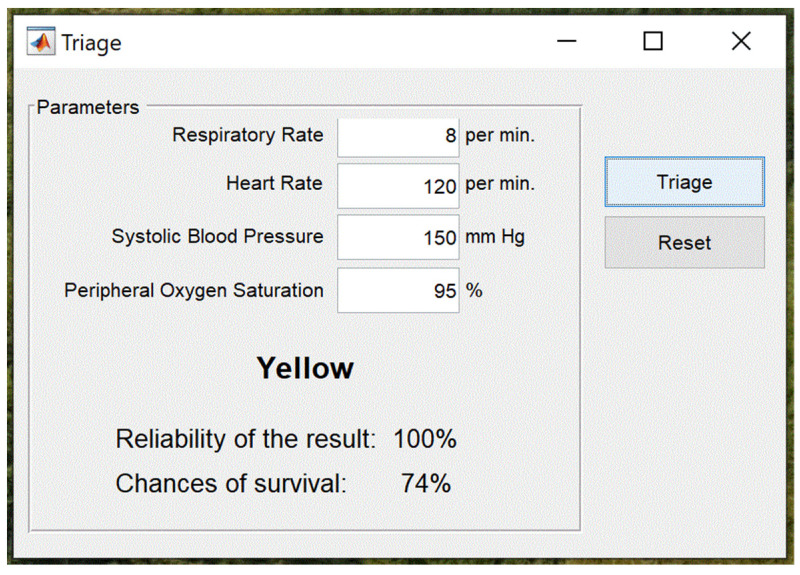
Example window with suggested triage color generated by AIS algorithm.

An example window with suggested decision on the triage visualization for selected soldier is shown in Figure 13.

Based on the parameters delivered to the AIS, the algorithms suggested that soldier should be tagged by the (yellow) color. All the vital signs are accessible to the AIS; thus, the reliability of the results is 100%. The SVM network suggests that chance of survival of the soldier is 74%. The final decision on the medical evacuation and the triage is now taken by medical personnel, who can additionally check the required biomedical signals of the soldier.

## 7. Visualization Module for Medical Personnel and Commanders

**Figure 14:**
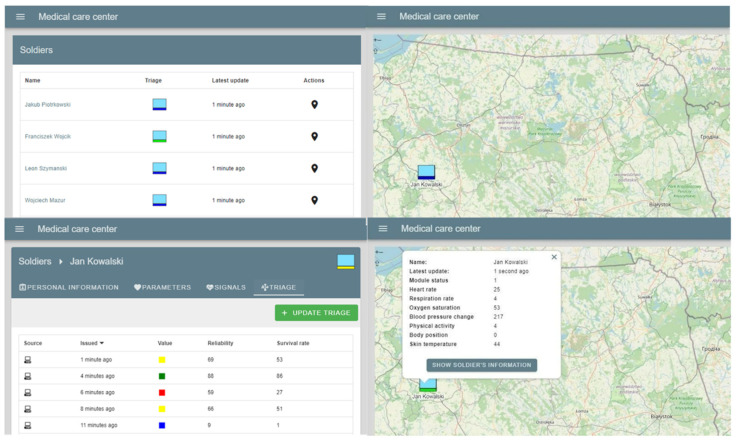
MSG Portal: view adapted to the T5” TRYTON Tactical Computer Terminal.

In order to visualize the collected data for operators of Medical Support Groups (MSG)/Battlefield Medical Monitoring Centre (BMMC), the dedicated portal was designed and implemented as a part of the Polish headquarter management system (HMS C3IS JASMINE) [29]. The portal provides access to information about soldiers monitored by the system. The MSG Portal view adapted to the T5” TRYTON Tactical Computer Terminal is shown in Figure 14. The main view presents basic data allowing medical personnel or commander to be oriented in a battlefield situation: a list of soldiers, the latest outcome of the triage and information about data updates. 

Using the software, the MSG operator has access to the detailed data of each monitored soldier. The portal provides obtaining the following data:

- Basic information: sex, birth date, weight, and the latest heart rate and blood pressure information;
- Status of vital signs, i.e.: respiratory rate, oxygen saturation, temperature and information on physical activity and body position;
- A dynamically updated graph reflecting changes in selected vital signs over time;
- Triage results: those read from the analysis and inference module (initial triage) and those entered manually by the operator or medical personnel (final triage).

The “Triage” tab presents a list of results read from the analysis and inference services. Medical Support Group Portal also allows visualization of the data using map layers providing rescuers with greater situational awareness and, thereby, facilitating planning for the prospective evacuation of victims. The symbology used as basic information indicates the triage result of each monitored soldier. In addition, operators can display a summary of the soldier’s last registered vital signs.

**Figure 15:**
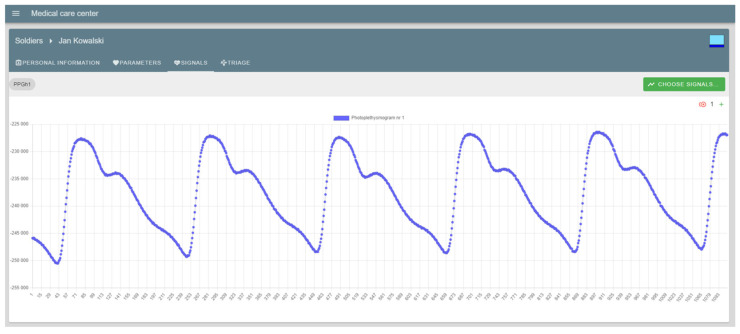
MSG Portal: visualization of an example biomedical signal (soldier name: Kowalski, signal name: Photoplethysmogram nr 1).

The “Signals” tab allows visualization of the selected biomedical signal registered by the measurement module of selected soldier. Figure 15 shows an example visualization of one of the testing biomedical signal (i.e., photoplethysmogram). 

The MSG portal was designed using responsive view technology that allows the interface to adapt to screen resolution and orientation. This allows efficient work also with mobile devices, e.g., TRYTON’s “T5 Tactical Computer Terminal”, which is adapted to work in any terrain, under various environmental conditions.

## 8. Conclusions

Currently, the DSS-MEDEVAC system was prepared in a form of the integrated testbed, where all the elements were implemented and tested. The tests confirmed that prepared sensors measured both vital signs and biomedical signals in acceptable form, required by the analysis and inference subsystem and by medical personnel. The biomedical signals were compared with the data obtained by professional medical equipment. The rescuers assessed that they can be very useful for remote health monitoring of wounded soldiers and are a good supplement to the information on triage suggested by the DSS-MEDEVAC system. The system efficiency will next be assessed in field tests to confirm all the required capabilities.

## Figures and Tables

**Table 1:** List of abbreviations used during DSS-MEDEVAC system description.

Abbreviation	Meaning
AAMB	Air Ambulance
AFE	Analog Front End
AIC	Analysis and Inference Capability
AIS	Analysis and Inference Subsystem
AMB	Ambulance
BAN	Body Area bio-sensors’ Network
BMMC	Battlefield Medical Monitoring Centre
BMS	Battlefield Management System
BodyPos	Body Position
C3IS	Command, Control, Communication, Intelligence and Surveillance
CN	Commander Node
CPU	Central Processing Unit
DMN	Decision Making Node
DTC	Data Transmission Capability
DTS	Data Transmission Subsystem
ECG	Electrocardiogram
gRPC	Google Remote Procedure Calls
HMS	Headquarters Management System
HR	Heart Rate
IP	Internet Protocol
MDTP	DSS-MEDEVAC Data Transmission Protocol
MEG	Medical Evacuation Groups
MEN	Medical Evacuation Node
MMC	Medical Monitoring Center
MPA	Mean Physical Activity
MSAC	Medical Situation Awareness Capability
MTF	Medical Treatment Facility
NAFv4	NATO Architecture Framework version 4
NVIC	Nested Vectored Interrupt Controller
PAS	Physical Activity Signal
PAT	Pulse Arrival Time
POI	Point of injury
PPG	Photoplethysmogram
PTT	Pulse Transit Time
RES	Respiratory Rate Signal
RR	Respiratory Rate
SBC	Single Board Computer
SN	Soldier Node
SpO_2_, SpO2	Oxygen Saturation
SVM	Support Vector Machine
TCCC	Tactical Combat Casualty Care
UART	Universal Asynchronous Receiver-Transmitter
USB	Universal Serial Bus
SBP	Systolic Blood Pressure
VCP	Virtual COM Port

## Data Availability

Not applicable.
